# XGR software for enhanced interpretation of genomic summary data, illustrated by application to immunological traits

**DOI:** 10.1186/s13073-016-0384-y

**Published:** 2016-12-13

**Authors:** Hai Fang, Bogdan Knezevic, Katie L. Burnham, Julian C. Knight

**Affiliations:** Wellcome Trust Centre for Human Genetics, University of Oxford, Oxford, OX3 7BN UK

**Keywords:** Software, eXploring Genomic Relations, Genomic summary data, Enhanced interpretation, Network analysis, Enrichment analysis, Similarity analysis, Annotation analysis

## Abstract

**Background:**

Biological interpretation of genomic summary data such as those resulting from genome-wide association studies (GWAS) and expression quantitative trait loci (eQTL) studies is one of the major bottlenecks in medical genomics research, calling for efficient and integrative tools to resolve this problem.

**Results:**

We introduce eXploring Genomic Relations (XGR), an open source tool designed for enhanced interpretation of genomic summary data enabling downstream knowledge discovery. Targeting users of varying computational skills, XGR utilises prior biological knowledge and relationships in a highly integrated but easily accessible way to make user-input genomic summary datasets more interpretable. We show how by incorporating ontology, annotation, and systems biology network-driven approaches, XGR generates more informative results than conventional analyses. We apply XGR to GWAS and eQTL summary data to explore the genomic landscape of the activated innate immune response and common immunological diseases. We provide genomic evidence for a disease taxonomy supporting the concept of a disease spectrum from autoimmune to autoinflammatory disorders. We also show how XGR can define SNP-modulated gene networks and pathways that are shared and distinct between diseases, how it achieves functional, phenotypic and epigenomic annotations of genes and variants, and how it enables exploring annotation-based relationships between genetic variants.

**Conclusions:**

XGR provides a single integrated solution to enhance interpretation of genomic summary data for downstream biological discovery. XGR is released as both an R package and a web-app, freely available at http://galahad.well.ox.ac.uk/XGR.

**Electronic supplementary material:**

The online version of this article (doi:10.1186/s13073-016-0384-y) contains supplementary material, which is available to authorized users.

## Background

One of the defining characteristics of medical genomics research is the large volume of genomic data available but the comparatively limited amount of biological knowledge revealed. This ‘big-data-limited-knowledge’ discrepancy stems from the heterogeneous forms and handling of raw data (usually unstructured), but is also attributed to imprecision in downstream interpretation [1, 2]. Data ready for downstream interpretation can be conveniently expressed as ‘genomic summary data’; that is, a list of genes or SNPs (or, more generally, genomic regions) along with summary statistics regarding the significance level (e.g. *p* values).

Using genomic summary data as a starting point for knowledge discovery is appealing. Cases in point are genome-wide association studies (GWAS) producing summary data on disease-associated genetic variants (GWAS SNPs) and expression quantitative trait loci (eQTL) mapping producing summary data on expression-associated genetic variants (eQTL SNPs). Firstly, it simplifies raw data (usually complex) and captures the essential information content. Secondly, GWAS and eQTL summary data are publicly available and well curated in relational databases, such as the GWAS Catalog [3], ImmunoBase [4], GTEx Portal [5], and Blood eQTL browser [6]. By comparison, the limited availability of genotyping data makes it prohibitively hard for ordinary users to conduct cross-disease and cross-study analyses, particularly those involving multiple data providers. Thirdly, cross-disease GWAS summary data hold great promise in understanding the genetic basis of disease comorbidity [7], whilst eQTL summary data could be useful in identifying genetic targets for drug development [8, 9].

Despite the availability and potential utility of this summary data, precise knowledge discovery itself is not trivial. It raises two critical issues: first, how to more systematically use widely distributed knowledge about genes and SNPs, much of which is unfortunately recorded in natural language; and second, how to achieve insights at the gene network level, which is desirable considering the interdependent and often synergistic nature of biological systems involving multiple players to complete the same task.

Knowledge use and access via ontologies provides an effective and efficient solution to the first issue. Using ontologies to annotate genes and gene products dates back to the beginning of this century when the Gene Ontology (GO) consortium initiated efforts to digitise gene functions [10]. Since then, a number of ontologies have been created to describe genes from the perspective of other knowledge domains (e.g. diseases [11] and phenotypes [12, 13]) and to describe protein domains [14]. Recent years have seen the shift in focus from the gene level to the SNP level (and generally to the genomic region level), accelerated by efforts to understand regulatory variants that most commonly underlie GWAS [15], resulting in the generation of increasing amounts of functional genomic data [16]. Compared to coding genes, which are well annotated by ontologies, non-coding genomic regions are lacking such annotations. Their interpretation relies heavily on either extrapolation from nearby genes or functional genomic data generated experimentally by large consortia such as ENCODE [17], FANTOM5 [18], BLUEPRINT Epigenome [19], TCGA [20], and Roadmap Epigenomics [21].

To address the second issue, gene interaction data should ideally be generated experimentally for every tissue, in both normal and diseased conditions given the fact that gene interactions are highly context-specific. In reality, an achievable alternative to this is to assimilate available context-specific interactions into a less context-specific, so-called ‘ground-truth gene network’ representing unified interaction knowledge. This strategy can be seen in databases such as STRING [22] and Pathway Commons [23]. Acting as a ‘scaffold’, the ground-truth gene network can then be integrated with context-specific summary data to identify the subset of the gene network, or ‘gene subnetwork’, that best explains that data.

The above issues identify an emerging need for ‘enhanced interpretation’ (effectiveness, efficiency, and transparency), particularly at the SNP and genomic region level. To meet this need, and also within our vision of its general use in e**X**ploring **G**enomic **R**elations, we develop the open-source software ‘XGR’ for enhancing knowledge discovery from genomic summary data. In addition to its comprehensive use of ontology and network information, we also show the uniqueness of XGR in 1) ontology tree-aware enrichment and similarity analysis and 2) cross-disease network and annotation analysis. Using real datasets [4, 24], we showcase its analytic power in uncovering the genetic landscape of immunological disorders based on GWAS summary data, and also demonstrate its added value in interpreting eQTL summary data of an immune-activated system. In short, XGR is software designed for enhanced interpretation necessary for doing big data science in genomics.

## Implementation

### Overview

**Fig. 1:**
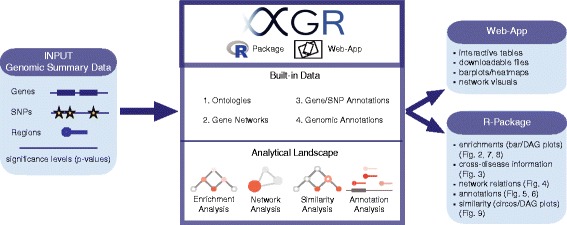
Schematic workflow of XGR: achieving enhanced interpretation of genomic summary data. This flowchart illustrates the basic concepts behind XGR. The user provides an input list of either genes, SNPs, or genomic regions, along with their significance levels (collectively referred to as genomic summary data). XGR, available as both an R package and a web-app, is then able to run enrichment, network, similarity, and annotation analyses based on this input. The analyses themselves are run using a combination of ontologies, gene networks, gene/SNP annotations, and genomic annotation data (built-in data). The output comes in various forms, including bar plots, directed acyclic graphs (DAG), circos plots, and network relationships. Furthermore, the web-app version provides interactive tables, downloadable files, and other visuals (e.g. heatmaps)

Figure 1 gives an overview of what XGR is and what the user can expect from it. XGR has two ends, the backend (an R package) [25] and the frontend (a web-app) [26]. Metaphorically, it works as a knowledge-driven ‘megabus’, carrying the passengers (users of varying computational skills) from the departure (a user-input list of genes, SNPs, or genomic regions) to the destination (outputs in a user-friendly format including ontology enrichments and network relationships). The petrol used by this megabus is the ontology and network knowledge (see next section), and the engine is its analytical capability, currently supporting enrichment, similarity, network, and annotation analysis (summarised in Table 1; see below for details). Put simply, XGR is designed to interpret genomic summary data resulting from modern genetic studies (differential expression, GWAS, and eQTL mappings), not targeting the upstream generation of summary data but instead enhancing its downstream biological discovery.

**Table 1:** A summary of XGR characteristics for tasks achieved and runtime required

Functions	Tasks achieved	Runtime^a^
*Enrichment analysis*		
xEnricher	A template for enrichment analysis	~40
xEnricherGenes	Gene-based enrichment analysis using a wide variety of ontologies^b^	~40
xEnricherSNPs	SNP-based enrichment analysis using Experimental Factor Ontology on GWAS traits	~70
xEnricherYours	Custom-based enrichment analysis using user-defined ontologies	~5
xEnrichConciser	Removing redundant ones from enrichment outputs	~15
xEnrichBarplot	Barplot of enrichment outputs	<1
xEnrichCompare	Side-by-side barplots of comparative enrichment outputs	<1
xEnrichDAGplot	DAG plot of enrichment outputs	<1
xEnrichDAGplotAdv	DAG plot of comparative enrichment outputs	<1
*Annotation analysis*		
xGRviaGeneAnno	Annotation analysis using nearby gene annotations by a wide variety of ontologies^b^	~60
xGRviaGenomicAnno	Annotation analysis using a wide variety of genomic annotations^c^	~30
*Similarity analysis*		
xSocialiser	A template for similarity analysis	~60
xSocialiserGenes	Gene-based similarity analysis using structured ontologies on functions, diseases, and phenotypes	~70
xSocialiserSNPs	SNP-based similarity analysis using Experimental Factor Ontology on GWAS traits	~60
xCircos	Circos plot of similarity outputs	~10
xSocialiserDAGplot	DAG plot of one set of terms used for similarity analysis	<1
xSocialiserDAGplotAdv	DAG plot of two sets of terms used for similarity analysis	<1
*Network analysis*		
xSubneterGenes	Gene-based network analysis	~60
xSubneterSNPs	SNP-based network analysis	~60
xVisNet	Network visualisation	<1

^a^Runtime (measured by seconds) tested using one core on Mac OS X

^b^Including structured ontologies on functions, diseases, and phenotypes, and non-structured ontologies on pathways, regulatory/expression signatures, druggability, structural domains, GTEx eGene tissues, others

^c^Including genomic annotations sourced from ENCODE, FANTOME5, BLUEPRINT Epigenome, Roadmap Epigenomics, The Cancer Genome Atlas, UCSC, others

### Source data and uniform representations

As a central part of the knowledge-driven interpretations, we have assembled currently available knowledge at the gene, SNP, and genomic region level (detailed below). All source data are represented uniformly as well-documented RData-formatted files, taking advantage of the R software open-development environment and its infrastructure packages such as igraph [27] and GenomicRanges [28]. The primary source data are maintained as part of in-house relational databases, from which Perl scripts are used to create RData files. Following an established pipeline, they are subject to regular updates and are also regularly supplemented to keep pace with the explosive nature of big data in genomics.

#### Ontologies and annotations at the gene level

Conceptually similar to a dictionary, an ontology contains well-defined vocabularies (called ‘terms’) and their relationships to each other, and is readable by both humans and computers. Depending on how relationships between terms are organised, ontologies can be broadly categorised into two types: 1) structured ontologies, where terms are organised in a tree-like structure (specifically a directed acyclic graph (DAG)), e.g. Gene Ontology [10], Disease Ontology [11], Phenotype Ontologies in human and mouse [12, 13]; 2) non-structured ontologies, where terms are simply listed as keywords, such as a collection of pathways from MSigDB [29], and of gene druggable categories from DGIdb [30]. Using ontologies to annotate genes is one of the most effective and scalable ways of capturing a particular knowledge sphere. The reuse of existing knowledge through ontology annotations is one of the key principles behind XGR. At the time of writing (October 2016), XGR supports nearly 30 gene annotations covering almost every type of knowledge domain, ranging from functions to diseases, phenotypes, pathways, and many others (Table 1). Whether structured or non-structured (in which case an artificial root is created to link together all terms), an ontology together with annotations is universally represented as an annotated directed graph. This design aids in performing operations such as graph visualisation, annotation propagation (according to the true-path rule), and semantic similarity calculations between terms. Ontologies and their identifier codes used in XGR are summarised in [31].

#### Ontology annotations at the SNP level

SNP annotations are based on the Experimental Factor Ontology (EFO). EFO standardises GWAS traits from the NHGRI GWAS Catalog using well-defined terms [3]. SNPs associated with one or more related traits grouped together by an EFO term are annotated by this term. Like any structured ontology, EFO is organised as a DAG. By the true-path rule, an SNP associated with a trait (mapped to an EFO term) should also be annotated by its ancestor terms (more general terms). For example, SNPs annotated by a term ‘EFO:0000540’ (immune system disease) consist of: 1) SNPs directly annotated with this term; and 2) SNPs associated with its child terms such as ‘EFO:0005140’ (autoimmune disease) and ‘EFO:0000706’ (spondyloarthropathy), which inherit the parent annotation. The problem of linkage disequilibrium (LD) makes it necessary to also include additional SNPs that are in strong LD with GWAS lead SNPs. For ease use in XGR, LD SNPs are pre-calculated using PLINK [32] based on the 1000 Genomes Project data [33] in different population panels, and those with R^2^ > 0.8 with GWAS lead SNPs are retained.

#### Annotations at the genomic region level

Unlike coding genes that are well annotated using ontologies, non-coding genomic regions lack such annotations. Interpretation of these regions relies largely on functional genomic data generated experimentally and on comparative genomic data predicted by computational methods. Genomic annotations currently supported in XGR include a broad spectrum of genomic and epigenomic data including, transcription factor binding sites, DNaseI hypersensitivity sites, histone modifications, expressed enhancers, and genome segmentations (Table 1). Each genomic annotation set is represented as a ‘GRanges’ object, primarily based on the ‘hg19’ (GRCh37) genome build. Also supported is conversion of genomic regions between commonly used builds: ‘hg19’, ‘hg38’ (GRCh38), and ‘hg18’. Data types, sources, and identifier codes used in XGR are summarised in [31].

#### Interaction networks at the gene level

XGR supports networks of different interaction types (functional, physical, and pathway-derived), of varying interaction quality (highest, high, and medium), and of two interaction directions (directed versus undirected). Networks are mainly sourced from the STRING database [22] and the Pathway Commons database [23]. STRING is a meta-integration of undirected interactions from a functional aspect, while Pathway Commons contains both undirected and directed interactions from a physical and pathway aspect. Interaction type and quality, as well as identifier codes used in XGR, are summarised in [31].

### Enrichment analysis

Enrichment analysis (or ‘Enricher’) is based on conventional statistical tests (Fisher’s exact test, hypergeometric or binomial test) to identify enriched ontology terms using either built-in or custom ontologies. The Fisher’s exact test establishes the independence between, for example, a user-defined gene group and a group of genes annotated by a term, and compares sampling only to the left part of the null background (without replacement). The hypergeometric test is to sample at random (without replacement) from the null background containing annotated and non-annotated genes. Finally, and in contrast to the hypergeometric test, the binomial test is to sample at random (with replacement) from the null background with the constant probability. As to the ease of reporting the significance level of a term (Additional file 1), they are, in order: hypergeometric test > Fisher’s exact test > binomial test. In other words, in terms of the calculated *p* value, hypergeometric test < Fisher’s exact test < binomial test. To further investigate the property of the statistical test, we simulated a random set of genes (having the same number of genes as in the real data) and estimated how often each enriched term in the real data would be expected from a null distribution based on the simulated data. As seen in Additional file 2, the chance (false positive rate) of enrichments in the real data that is falsely called significant from the simulated null data is extremely low. We also assessed false positive rate by simulating a random set of genes of different sizes and found they were independent of the size of gene sets (Additional file 3).

**Fig. 2:**
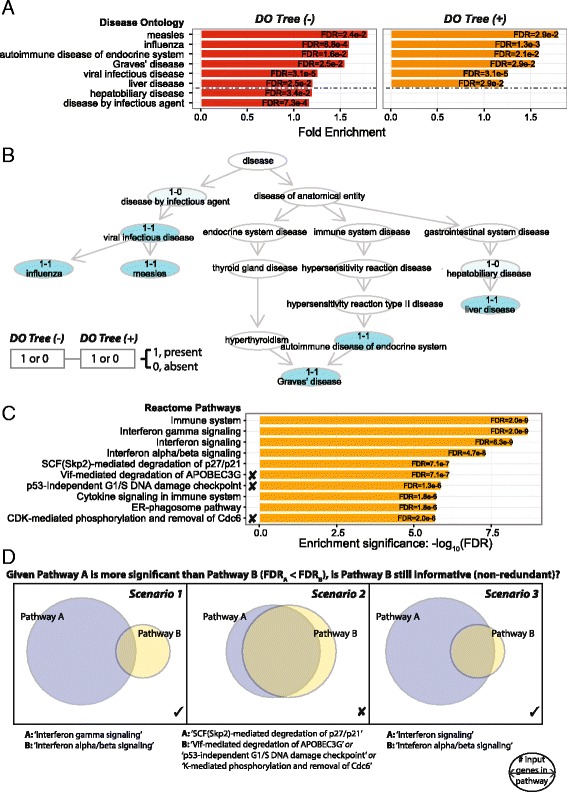
Necessity of respecting ontology tree-like structure and of removing redundant non-structured pathways in enrichment analysis. This is demonstrated by analysing differentially expressed genes induced by 24-h interferon gamma in monocytes. The effect of taking ontology tree-like structure into account is demonstrated using Disease Ontology (DO) and the removal of redundant non-structured ontologies using Reactome pathways. **a** Side-by-side bar plots comparing the significant DO terms between the analysis without considering the tree structure (*DO Tree(-)*) versus the analysis considering the tree structure (*DO Tree(+)*). The horizontal dotted line separates commonly identified terms (*top section*) and redundant terms in the *DO Tree(-)* analysis. **b** DAG plot comparing commonly identified terms (coloured in *cyan*) and redundant terms from the *DO Tree(-)* analysis (coloured in *light cyan*). The term name (if significant) is prefixed in the form ‘x1-x2’. x1 represents ‘*DO Tree (-)*’ and x2 ‘*DO Tree (+)*’. The value of x1 (or x2) can be ‘1’ or ‘0’, denoting whether this term is identified (present) or not (absent). **c** The top pathway enrichments, with the redundant pathways to be removed indicated (***X***). **d** Illustrations of whether a less significant pathway B is redundant considering a more significant pathway A. Pathway B is counted redundant if it meets both criteria. Criterion 1: more than 90% of input genes annotated with pathway B are also covered by pathway A. Criterion 2: more than 50% of input genes annotated with pathway A are also covered by pathway B. Scenario 1 does not meet either criteria, scenario 2 meets both, and scenario 3 meets criterion 1 but not criterion 2. Notably, criterion 2 ensures the resulting pathways (as shown in scenario 3) are informative in capturing knowledge spheres of different granularities; otherwise, pathway B would be considered redundant in scenario 3, leading to loss of information. *FDR*: false discovery rate

XGR is unique in being designed to produce much more informative enrichment results. This is achieved either by taking into account the ontology tree-like structure when using a structured ontology or by applying a filtering procedure when using a non-structured ontology (Fig. 2).

#### Using a structured ontology

The basic idea is to account for the dependency of terms during enrichment analysis; for example, estimating the significance of a term after removing gene annotations that its significant child terms have. For technical details, please refer to publications [34, 35].

#### Using a non-structured ontology

A filtering procedure is applied to further remove redundant terms resulting from enrichment analysis. Take pathway enrichment analysis as an example (Fig. 2), assuming that there are two significant pathways, A and B, and that pathway A is more significantly enriched than pathway B. The less significant pathway B is deemed to be redundant if it meets both of the following criteria: 1) >90% of input genes annotated with pathway B are also annotated by pathway A; and 2) >50% of input genes annotated by pathway A are also annotated by pathway B. Both criteria were chosen empirically, as we observed that the increase in criterion 1 (90%) would result in the inability to remove redundant terms (Additional file 4a) and that criterion 2 (50%) produces the relative stability of redundant terms being removed (Additional file 4b). It should be noted that, although these default criteria should be applicable in most circumstances, the user can refine them by manipulating different thresholds.

#### Functionality

The function ‘xEnricherGenes’ conducts gene-level enrichment analysis using either structured ontologies or non-structured ontologies. The function ‘xEnricherSNPs’ conducts EFO-based enrichment analysis at the SNP level, allowing the inclusion of additional SNPs that are in LD with input SNPs. The function ‘xEnricherYours’ enables customised analysis using the user’s own ontologies and annotations for entities beyond genes and SNPs. Enrichment outputs are stored as an object of a newly defined class ‘eTerm’. Directly operating on this object, the function ‘xEnrichBarplot’ visualises enrichment results using a barplot, and the function ‘xEnrichDAGplot’ uses a DAG plot to display enriched terms in the context of the ontology tree. The function ‘xEnrichCompare’ is specially designed for side-by-side barplot comparison when involving two or more enrichment results (e.g. across different conditions but using the same ontology). The function ‘xEnrichDAGplotAdv’ takes this comparison further, highlighting which terms are shared and which are unique in the ontology tree.

### Annotation analysis

Annotation analysis (or ‘Annotator’) aims to interpret a list of user-defined genomic regions in two ways: either via annotations of nearby genes by ontologies or via co-localised functional genomic annotations. Thanks to the diversity of source data available and the generalisation of data representation (see above), XGR enables multifaceted interpretation of poorly annotated genomic regions.

#### Functionality

The function ‘xGRviaGeneAnno’ takes as input a list of user-defined genomic regions, defines the nearest genes within a user-specified distance gap, and conducts enrichment analysis using nearby gene annotations. Similar to enrichment analysis at the gene level, this function gives the choice of structured and non-structured ontologies, producing informative enrichment results that can be visually displayed/compared. Alternatively, both functions ‘xGRviaGenomicAnno’ and ‘xGRviaGenomicAnnoAdv’ conduct region-based enrichment analysis using co-localising functional genomic annotations. The function ‘xGRviaGenomicAnno’ uses the binomial test for estimating the significance of overlaps at base resolution. The function ‘xGRviaGenomicAnnoAdv’ estimates the significance of the observed overlaps against the expectation under the null distribution, which is generated through random sampling from background genomic regions. By default, the background uses annotatable genomic regions (depending on which genomic annotations are used). However, it is advisable for the user to specify this background according to experimental settings. Enrichment results (as ‘eTerm’ objects) from annotation analysis can be visualised and compared using functions ‘xEnrichBarplot’ and ‘xEnrichCompare’.

### Similarity analysis

Similarity analysis (or ‘Socialiser’) calculates semantic similarity between two genes (or between two SNPs) based on their ontology annotation profiles. More precisely, it assesses the degree of relatedness in meaning of annotation profiles from a structured ontology. The function ‘xSocialiserGenes’ conducts similarity analysis for genes using annotations by structured ontologies, while the function ‘xSocialiserSNPs’ conducts SNP-based similarity analysis using annotations from EFO.

#### SNP semantic similarity

The procedure used to calculate semantic similarity between two SNPs is as follows. First, the information content (IC) of a term is defined to measure how informative it is when used to annotate SNPs: –log_10_(frequency of SNPs annotated by this term). Semantic similarity between each pair of terms is pre-calculated, usually quantified as IC at the most informative common ancestor (MICA) of the two terms. Finally, semantic similarity *SIM(S*_*1*_ *, S*_*2*_ *)* between two SNPs, *S*_*1*_ and *S*_*2*_, is derived from pairwise term similarity, using best-matching (BM) based methods: average (Eq. 1), maximum (Eq. 2), or complete (Eq. 3). For a term in the annotation profile of one SNP, all these BM-based methods calculate the maximum similarity to any term in the profile of the other SNP. It can be deduced from the formula that the average and maximum methods are more sensitive to the number of terms than the complete method. However, due to the current sparse nature of EFO-based annotation of GWAS SNPs, using any of the three methods produces similar results. Indeed, they are interchangeable, although results from the average and maximum methods are more similar to each other than to the complete method (Additional file 5). By default, the complete method is used to minimise the impact of the number of terms. The resulting SNP semantic similarity network is a weighted undirected graph, with SNPs as nodes and semantic similarity scores as the edge weights. Inclusion of LD SNPs is also possible for similarity analysis.

#### Basis of SNP similarity

The function ‘xCircos’ displays the similarity results using a circos plot, in which the degree of similarity between two SNPs is indicated by the coloured link. This function can be used to display the most similar links, or those links involving a specific SNP only. Two functions, ‘xSocialiserDAGplot’ and ‘xSocialiserDAGplotAdv’, are specially designed to explore the basis of similarity seen in the circos plot. The function ‘xSocialiserDAGplot’ is used to visualise the ontology annotation profile for an SNP, i.e. as a DAG plot of terms used to annotate the SNP, including original annotations (rectangular nodes) and inherited annotations (elliptical nodes). The function ‘xSocialiserDAGplotAdv’ uses a DAG plot to compare annotation profiles between two similar SNPs.

1 $$ SIM\left({S}_1,{S}_2\right)=\frac{1}{2}\times \left(\frac{1}{n_1}\sum_{t_1\in {T}_1}\underset{t_2\in {T}_2}{MAX}\left( MICA\left({t}_1,{t}_2\right)\right)+\frac{1}{n_2}\sum_{t_2\in {T}_2}\underset{t_1\in {T}_1}{MAX}\left( MICA\left({t}_1,{t}_2\right)\right)\right), $$

2 $$ SIM\left({S}_1,{S}_2\right)= MAX\left(\frac{1}{n_1}\sum_{t_1\in {T}_1}\underset{t_2\in {T}_2}{MAX}\left( MICA\left({t}_1,{t}_2\right)\right),\frac{1}{n_2}\sum_{t_2\in {T}_2}\underset{t_1\in {T}_1}{MAX}\left( MICA\left({t}_1,{t}_2\right)\right)\right), $$

3 $$ SIM\left({S}_1,{S}_2\right)=MIN\left(\underset{t_1\in {T}_1}{U}\underset{t_2\in {T}_2}{MAX}\left( MICA\left({t}_1,{t}_2\right)\right),\underset{t_2\in {T}_2}{U}\underset{t_1\in {T}_1}{MAX}\left( MICA\left({t}_1,{t}_2\right)\right)\right), $$

where *T*_*1*_ is a set of *n*_*1*_ EFO terms used to annotate *S*_*1*_, *T*_*2*_ is a set of *n*_*2*_ EFO terms annotating *S*_*2*_, *MICA(t*_*1*_ *, t*_*2*_ *)* is the IC of the MICA of two terms *t*_*1*_ and *t*_*2*_, operators *MAX*, *MIN*, and *U* denote, respectively, maximum, minimum, and union.

### Network analysis

Network analysis (or ‘Networker’) identifies the subset (gene subnetwork) from a gene interaction network with nodes/genes labelled with significance information. Depending on how the node/gene significance information is provided, there are two types of network analyses supported in XGR: gene-based network analysis and SNP-based network analysis.

#### Gene-based network analysis

The node/gene information is directly provided, e.g. differentially expressed genes with significance measured by false discovery rate (FDR). Given a gene interaction network with nodes/genes labelled with significance, the function ‘xSubneterGenes’ searches for a maximum-scoring gene subnetwork enriched with the most significant (highly scored) genes but allowing for a few less significant genes as linkers (usually hubs). The search for this maximum-scoring subnetwork is achieved via heuristically solving a prize-collecting Steiner tree problem; this approach has been demonstrated to be superior to other state-of-the-art methods. If required, an iterative procedure is applied to identify the subnetwork with a desired number of nodes/genes. For details please refer to our previous publication [36].

#### SNP-based network analysis

**Fig. 3:**
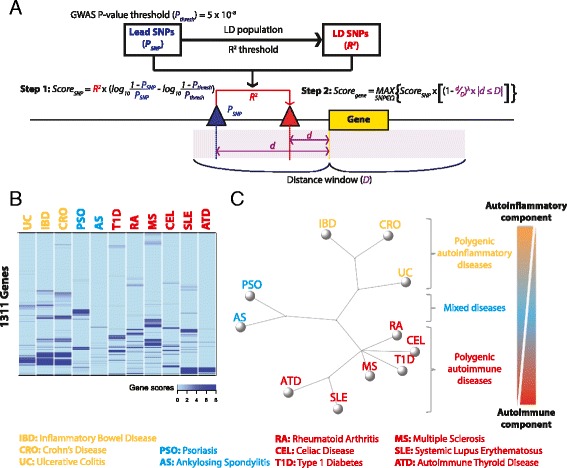
Informativeness of using cross-disease GWAS summary data in characterising relationships between immunological disorders. **a** Gene scoring from GWAS SNPs prior to network analysis. **b** Heatmap of cross-disease gene scores for 11 common immunological disorders based on ImmunoBase GWAS summary data. **c** Consensus neighbour-joining tree based on the gene-scoring matrix resolves disease classification/taxonomy according to the genetic and cellular basis of autoinflammation and autoimmunity. Subdivided into 1) polygenic autoinflammatory diseases with a prominent autoinflammatory component, 2) polygenic autoimmune diseases with a prominent autoimmune component, and 3) mixed diseases having both components. Inter-disease distance is defined as the cumulative difference in gene scores

We extend the network analysis to the SNP level, allowing node/gene information to be indirectly provided (i.e. derived from the input), e.g. via GWAS SNPs along with *p* values. The function ‘xSubneterSNPs’ is designed to identify a gene subnetwork that is likely modulated by input SNPs and/or their LD SNPs. It consists of three steps (Fig. 3a):

1. SNP scoring (Eq. 4), which considers the *p* values, the threshold (e.g. 5e-8 for typical GWAS), and (for LD SNPs) LD strength *R* ^*2*^.
2. Gene scoring (Eq. 5), which scores genes based on genomic proximity to quantify their genetic modulation by SNPs (and LD SNPs).
3. Network scoring, using the function ‘xSubneterGenes’ to identify a maximum-scoring gene subnetwork (with the desired number of nodes if required).

4 $$ Scor{e}_{SNP}={R}^2\times \left(lo{g}_{10}\frac{1-{P}_{SNP}}{P_{SNP}}-lo{g}_{10}\frac{1-{P}_{thresh}}{P_{thresh}}\right), $$

where *P*_*SNP*_ is the SNP *p* value, *P*_*thresh*_ is the significance threshold (usually 5e-8), and *R* ^*2*^ is the LD strength.

5 $$ Scor{e}_{gene}=\underset{SNP\in \varOmega }{MAX}\left\{ Scor{e}_{SNP}\times \left[{\left(1-\frac{d}{D}\right)}^{\lambda}\times \left|d\le D\right|\right]\right\}, $$

where *Score*_*SNP*_ is the SNP score calculated using Eq. 4, *d* is the gene-to-SNP distance within a maximum of the distance window *D*, *λ* is the decay exponent controlling the decaying influence of an SNP on a nearby gene as the distance increases, *Ω* stands for collections of SNPs (input SNPs and LD SNPs), and *MAX* denotes maximum scoring scheme used here to only keep the most-informative SNP when a large number of interdependent SNPs are located within the same genetic region.

### Other implementation issues

#### Control for multiple testing

Where a large number of tests are involved, we adjust *p* values either controlling the FDR (by default) or controlling the family-wise error rate (FWER). FDR is a less stringent condition than FWER. The user can choose how to account for multiple testing.

#### R package dependency

We rely on the package ‘ggplot2’ [37] for various visuals and adapt the package ‘RCircos’ [38] for a circos plot. Where necessary for high-performance parallel computing, two packages, ‘doMC’ and ‘foreach’, are used to reduce computational costs. Other dependent packages are listed in [25].

#### Web-app implementation

We use a next-generation Perl web framework ‘Mojolicious’ [39], under which the XGR web-app is portable requiring nearly zero-effort maintenance. Its maintenance is further simplified as the web-app is purely powered by the XGR R package (stably deposited into the CRAN repository).

## Results

We demonstrate the application of XGR to interpret three commonly encountered types of genomic summary data: 1) gene sets resulting from differential expression studies; 2) GWAS SNPs from GWAS summary data; and 3) eQTL SNPs from eQTL summary data. We first illustrate the functionalities supported in XGR to interpret differentially expressed genes induced by innate immune stimuli [24]. At the SNP level, we showcase the analytical power of XGR to interpret GWAS SNPs associated with immunological disorders [4] and to interpret eQTL SNPs relevant to immune-stimulated systems [24]. Within these showcases, we demonstrate improved performance compared to conventional analyses. All these comparisons and showcases are provided on the software website and are reproducible following step-by-step instructions [31].

### Interpreting summary data resulting from differential expression studies

This demo illustrates the power of XGR to interpret the output from differential expression studies, with the focus on how to carry out ontology-based enrichment analysis to achieve more informative results.

#### Necessity of respecting the ontology tree structure when using structured ontologies for enrichment analysis

We use Disease Ontology (DO) to interpret differentially expressed genes induced by 24-h interferon (IFN)-γ treatment of primary human monocytes [24]. Figure 2a shows side-by-side comparison of enrichment results with and without consideration of the ontology tree structure. As expected, both analyses identify a significant link between IFN-γ-induced transcriptome changes and genes involved in viral infectious disease (e.g. influenza and measles) and autoimmunity (e.g. Graves’ disease). However, considering the ontology tree structure allows exclusion of significant but less informative DO terms such as ‘disease by infectious agent’. This becomes clearer when visualising enriched terms in the context of the DO hierarchy (Fig. 2b), showing that the child term ‘viral infectious disease’ is a much more precise descriptor.

#### Necessity of filtering redundant terms when using non-structured ontologies for enrichment analysis

When using non-structured ontologies such as a collection of pathways, we develop a post-enrichment filtering procedure to identify redundant terms for removal (Fig. 2c). The goal is to filter out only pathways that have been covered by a more significant pathway of similar granularity (scenario 2 in Fig. 2d). However, if a pathway is informative in capturing specific knowledge and the more significant pathway is very general, XGR will retain it (scenario 3 in Fig. 2d). This ensures the resulting enrichments are non-redundant but still informative enough to help interpretation.

### Interpreting GWAS summary data

This demo showcases the power of XGR to interpret GWAS SNPs, including network and annotation analysis.

#### SNP-modulated genes and their informativeness for characterising disease relationships

Unique to XGR is its ability to identify SNP-modulated gene networks. To do this, XGR first defines and scores genes that are likely under the genetic influence of GWAS SNPs (Fig. 3a). When applied to GWAS summary data for 11 common immunological diseases (available from ImmunoBase [4]), we find that genes scored in this way (Fig. 3b) are able to resolve disease taxonomy, providing independent evidence for a proposed continuum of autoinflammation and autoimmunity [40]. As seen in the consensus neighbour-joining tree (Fig. 3c), the diseases analysed span an autoinflammatory–autoimmune spectrum, reflecting the relative roles of the innate immune response versus the adaptive immune response in disease development. The diseases analysed are divided into three categories: 1) polygenic autoinflammatory diseases with a prominent autoinflammatory component, including inflammatory bowel disease (IBD), Crohn’s disease (CRO), and ulcerative colitis (UC); 2) polygenic autoimmune diseases with a prominent autoimmune component, including celiac disease (CEL), autoimmune thyroid disease (ATD), type 1 diabetes (T1D), rheumatoid arthritis (RA), multiple sclerosis (MS), and systemic lupus erythematosus (SLE); and 3) mixed diseases having both components, including psoriasis (PSO) and ankylosing spondylitis (AS). Our analysis also shows that polygenic autoinflammatory diseases may be subdivided into two subtypes, one comprising SLE and ATD, the other CEL, MS, T1D, and RA.

#### SNP-modulated gene networks underlying disease categories

**Fig. 4:**
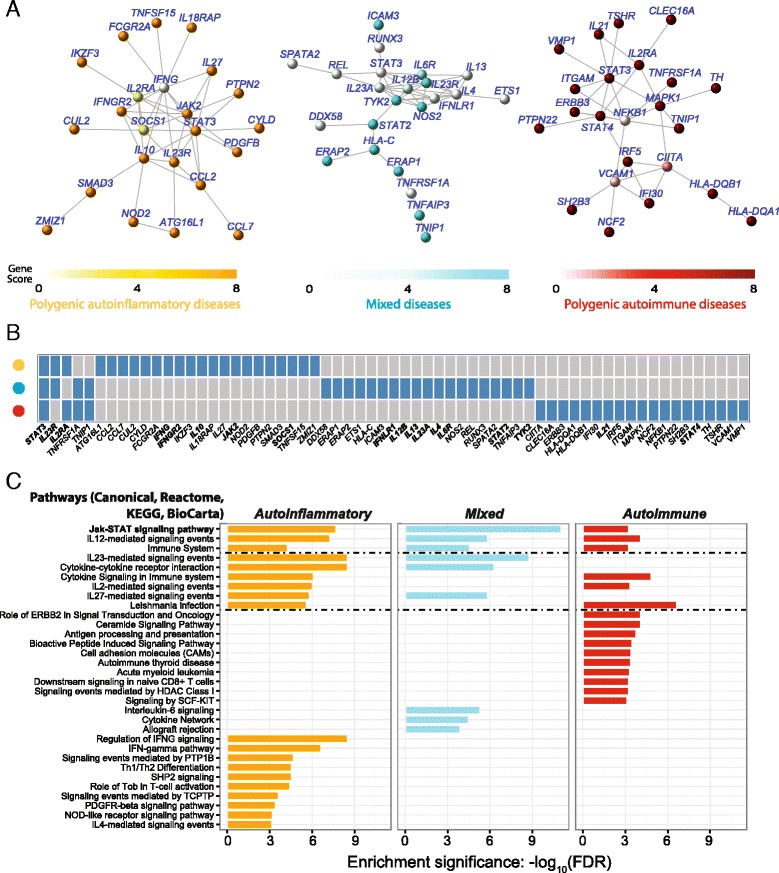
SNP-modulated gene networks underlying three immunological disease categories. **a** The top-scoring gene network for the three disease categories: autoinflammatory diseases (*orange*), mixed diseases (*cyan*), and autoimmune diseases (*red*). **b** Network genes shared by and unique to disease categories. Genes involved in the Jak-STAT signalling pathway are in *bold text*. **c** Pathway enrichment analysis of network genes using all pathway ontologies and eliminating redundant pathways. The *horizontal dotted line* separates pathways common to all three disease categories (*top section*; e.g. Jak-STAT signalling pathway), those shared by any two categories (*middle*), and those only enriched in one category (*bottom*)

To understand the molecular basis of the observed autoinflammatory–autoimmune disease continuum, we next identify the top SNP-modulated gene networks based on pooled GWAS SNPs for each of the three categories (Fig. 4a). The gene networks identified contain hallmark genes for each category, for example, *PTPN22* and *MHC* genes for polygenic autoimmune diseases and *NOD2* for polygenic autoinflammatory diseases. Comparing network genes identifies one gene, *STAT3*, common to all three categories; a few genes, including *TNFSF1A*, *TNIP1*, and two interleukin (IL) genes (*IL23R* and *IL2RA*) are shared by two categories, and many genes are unique to one group, suggesting that each disease category has its own specialised network architecture (Fig. 4b). However, at the pathway level we find much more commonality between categories (Fig. 4c). For instance, all groups share the Jak-STAT signalling pathway. In addition to the gene *STAT3*, each category has unique players in this pathway, including *IL2*, *IFNG*, *IFNGR2*, *IL10*, *JAK2*, and *SOCS1* in the polygenic autoinflammatory disease gene network, *IFNLR1*, *IL12B*, *IL13*, *IL23A*, *IL4*, *IL6R*, *STAT2*, and *TYK2* in the mixed disease gene network, and *IL21* and *STAT4* in the autoimmune gene network. The IL12-mediated signalling pathway is another pathway shared by all. These results suggest that targeting different members of the same pathway for treatment might be a useful approach. Among pathways shared by any two groups, we find the IL pathways are informative for shared disease features: the IL23 and IL27 pathways are common to both autoinflammatory and mixed diseases, while the IL2 pathway is common to both autoinflammatory and autoimmune diseases.

#### Functional and phenotypic annotation of genes harbouring GWAS SNPs for each of three disease categories

**Fig. 5:**
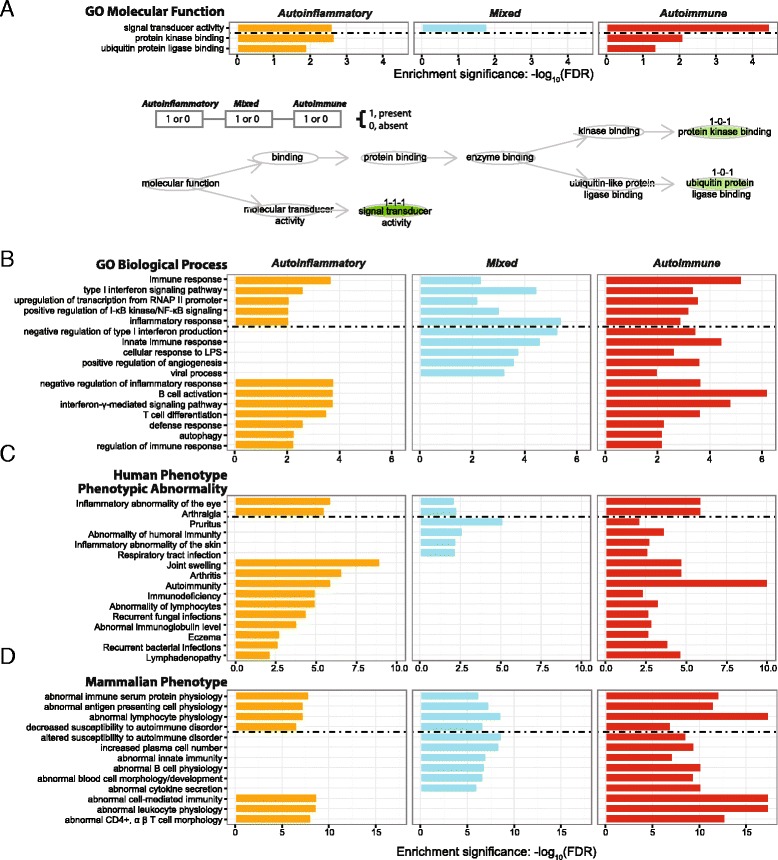
Functional and phenotypic annotation analysis of genes harbouring GWAS SNPs for three immunological disease categories. Visualised in aside-by-side bar plot and/or DAG plot using functional ontologies, including **a** GO molecular function and **b** GO biological process; and using phenotype ontologies in human and mouse, including **c** human phenotype phenotypic abnormality, and **d** mammalian phenotype

We use annotation analysis to interpret pooled GWAS SNPs for each of the three categories by looking directly at genes harbouring these SNPs. Here we focus on commonalities across two or three disease categories in terms of functions and phenotypes shared (Fig. 5). As shown in Fig. 5a, three disease groups share genetic variants in genes with signal transduction activity, and variants for both autoinflammatory and autoimmune diseases are enriched in genes with kinase and ubiquitin ligase binding activities. Similarly, functional commonalities can be identified using GO biological processes (Fig. 5b). Using phenotype annotations, XGR is able to reveal shared abnormal phenotypes both in human and mouse (Fig. 5c, d); they include diverse abnormalities relating to inflammation and immunity, consistent with the phenotypic complexity of these common disease categories.

#### Genetic and epigenetic characterisation of GWAS SNPs for each of three disease categories

**Fig. 6:**
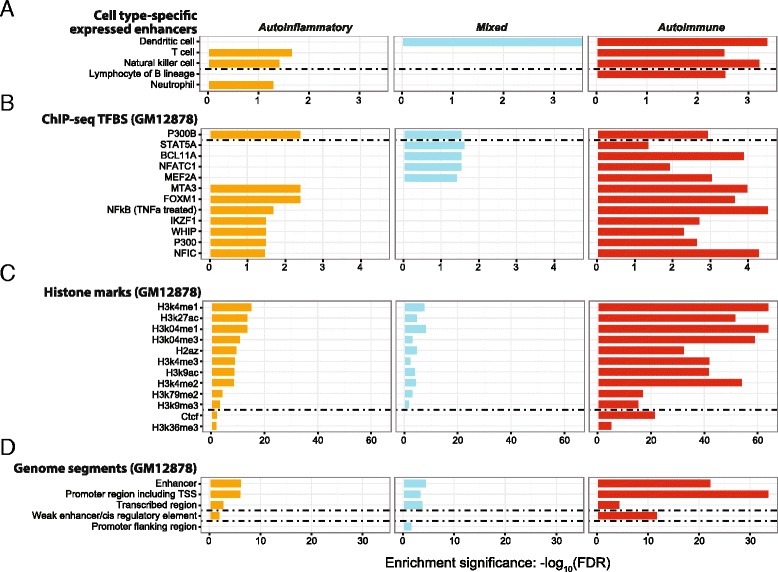
Functional genomic annotation analysis of GWAS SNPs by genomic location for three immunological disease categories. **a** Using the FANTOM cell type-specific expressed enhancer data, **b** using ENCODE ChIP-seq transcription factor binding site (*TFBS*) data, **c** using ENCODE histone mark data, and (**d**) using ENCODE genome segment information. Panels **b**–**d** use genetic and epigenetic data generated in the GM12878 lymphoblastoid cell line

Using functional genomic annotations supported in XGR, we are also able to compare and define characteristics underlying each of the three categories (Fig. 6). As a proof of principle, we use cell type-specific genetic and epigenetic annotations to characterise pooled GWAS SNPs per disease category. Based on cell type-specific expressed/active enhancers from FANTOM5 (Fig. 6a), SNPs for autoimmune diseases tend to be co-localised with expressed enhancers in B lineage lymphocytes, in dendritic cells (also seen with SNPs for mixed diseases), in T cells, and in natural killer cells (also in SNPs for autoinflammatory diseases). Co-localisation with expressed enhancers in neutrophils is only seen for autoinflammatory disease SNPs. Using genetic and epigenetic data generated in the GM12878 lymhoblastoid cell line (Fig. 6b–d), we identify common characteristics, including transcription factor binding sites, histone marks, and genome segments. The multiple layers of information revealed by XGR provide a powerful tool to characterise genomic features underlying disease categories.

### Interpreting eQTL summary data

This demo highlights the power of XGR to interpret eQTL SNPs, including enrichment and similarity analysis.

#### Performance comparisons between conventional enrichment analysis and ontology-based enrichment analysis

**Fig. 7:**
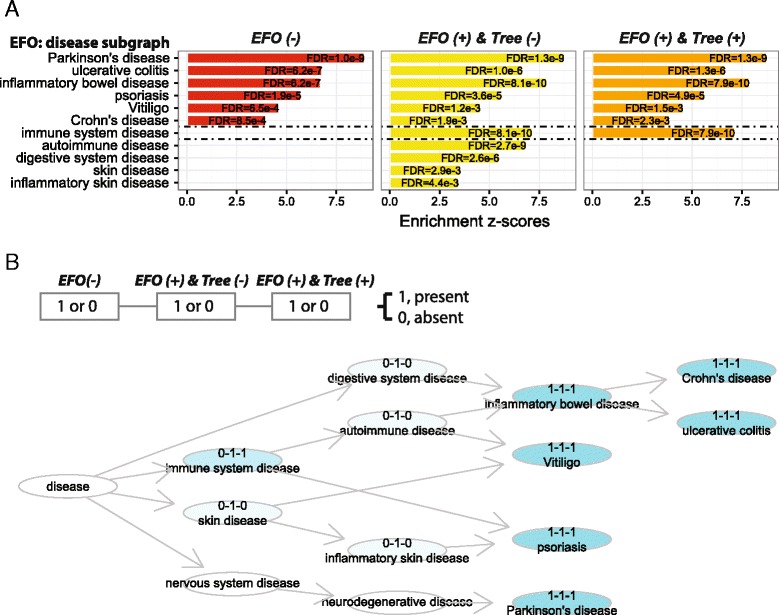
Necessity of using Experimental Factor Ontology and respecting ontology tree-like structure in SNP-based enrichment analysis. This is demonstrated using the disease subgraph of the Experimental Factor Ontology (*EFO*) and analysing *cis*-eQTLs induced by 24-h IFN-γ. **a** Side-by-side bar plots comparing the significant EFO terms between the analysis not using EFO (conventional analysis; *EFO (-)*) and two ontology-based analyses: the *EFO (+) & Tree (-)* analysis using EFO but without respecting the ontology tree, and the *EFO (+) & Tree (+)* analysis using EFO and also respecting the ontology tree. The *horizontal dotted lines* separate commonly identified terms (*top*), the terms unique to the ontology-based analyses (*middle*), and the redundant terms identified by the *EFO (+) & Tree (-)* analysis (*bottom*). **b** DAG plot comparing terms identified by all analyses (coloured in *cyan*), by two analyses (coloured in *light cyan*), and only by one analysis (coloured in *lightest cyan*). The term name (if significant) is prefixed in the form of ‘x1-x2-x3’. In this case, x1 for ‘*EFO (-)*’, x2 for ‘*EFO (+) & Tree (-)*’, x3 for ‘*EFO (+) & Tree (+)*’. The value of x1–3 can be ‘1’ or ‘0’, denoting whether this term is identified (present) or not (absent)

Conventionally, SNP-based enrichment analysis is only done using traits originally reported in GWAS. However, GWAS traits can be mapped onto EFO, enabling us to look at general terms (representing a group of related traits) and to include more annotated SNPs: GWAS-reported SNPs (‘original annotations’) and inherited SNPs from its child terms (‘inherited annotations’). By convention, SNP-based enrichment analysis considers LD SNPs. The benefit of using EFO and justification of our ontology tree-aware enrichment analysis is demonstrated using the disease part of EFO to interpret *cis*-eQTLs induced by 24-h IFN-γ treatment of human monocytes (Fig. 7a). We consider three scenarios: 1) ‘*EFO (-)*’ not using EFO (i.e. conventional analysis); 2) ‘*EFO (+) & Tree (-)*’ using EFO but without respecting the ontology tree; and 3) ‘*EFO (+) & Tree (+)*’ using EFO and also respecting the ontology tree. Using EFO identifies disease terms that would otherwise be missed with conventional analysis. However, without respecting the ontology tree, the redundant disease terms identified would become a burden for interpretation. Compared to conventional analysis, our ontology tree-aware analysis identifies an additional term (‘immune system disease’) that summarises the overall enrichments, illustrated by visualising the enrichment results in the EFO tree (Fig. 7b).

#### Cross-condition comparative enrichment analysis

**Fig. 8:**
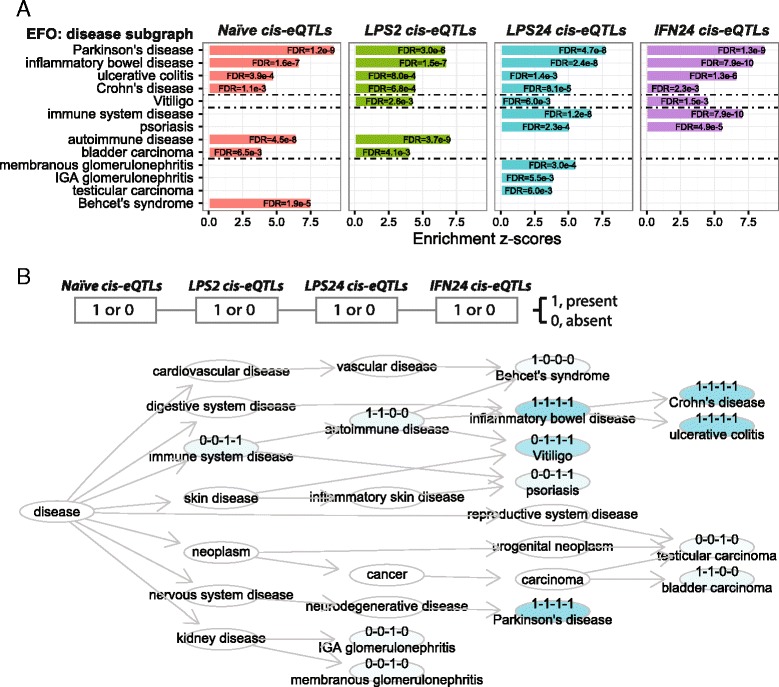
Comparative enrichment analysis for *cis*-eQTL SNPs under four immunologically relevant conditions. The four eQTL SNP sets are: naive state (*Naïve cis-eQTLs*), induced by 2-h LPS (*LPS2 cis-eQTLs*), by 24-h LPS (*LPS24 cis-eQTLs*), and by 24-h IFN-γ (*IFN24 cis-eQTLs*). All analyses are using the disease subgraph of EFO and respecting the ontology tree. **a** Side-by-side bar plots comparing the significant EFO terms across the four conditions. The *horizontal dotted lines* separate terms shared by four conditions (*top*), by three conditions (*upper middle*), by two conditions (*lower middle*), and unique to one condition (*bottom*). **b** DAG plot comparing the significant EFO terms across the four conditions. Nodes/terms are coloured according to the number of conditions sharing the terms

We previously reported context-specific induced *cis*-eQTLs that were frequently enriched for disease risk loci [24]. Using ontology tree-aware analysis, we re-interpret these context-specific eQTLs by comparing their disease associations. Side-by-side barplots together with tree-like DAG plots in Fig. 8 give sufficient information for straightforward interpretation, aiding in hypothesis generation. Induced *cis*-eQTLs, whether in the naïve state or upon immune stimulation, are consistently overrepresented in autoinflammatory diseases (IBD, CRO and UC) as expected, but also linked to Parkinson’s disease (PD).

#### SNP similarity analysis based on disease trait profiles

**Fig. 9:**
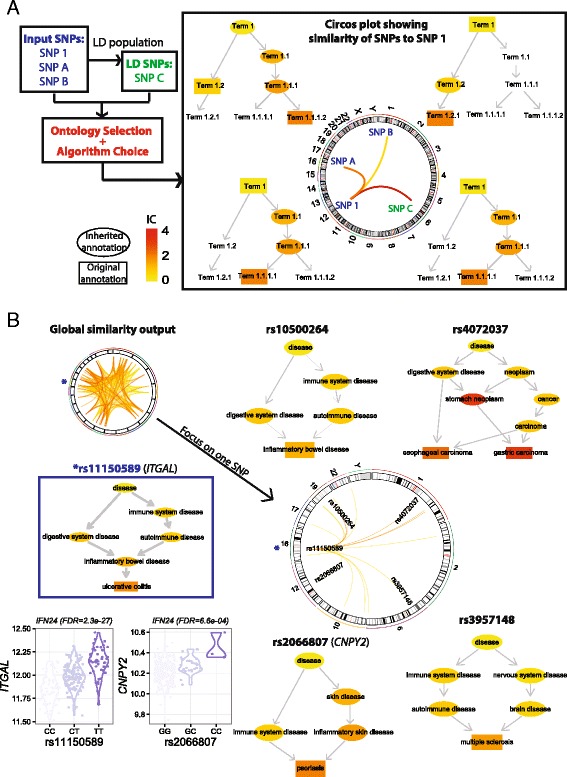
SNP similarity analysis interpreting eQTL SNPs. **a** This toy example illustrates the SNP similarity analysis, which calculates pairwise semantic similarity between SNPs using the Experimental Factor Ontology (EFO). The input is a list of SNPs, with the option to include SNPs in linkage disequilibrium (*LD*). The output is a circos plot, with the link line colour graded according to the degree of semantic similarity between each pair of SNPs. The calculation of similarity takes into account the annotation profile of the SNPs, the information content (IC) of the term, and the term–term similarity. In our example, each SNP is directly annotated by two terms, and inherit additional annotation terms according to the true-path rule. The terms are coloured according to their IC; original terms have a *rectangular border*, inherited terms an *elliptical border*. SNP 1 shows similarity of varying degrees to the other three SNPs based on their shared annotation profiles. SNP 1 and SNP C share both “Term 1” and the very informative “Term 1.1.1.1”; as such, they have a very high degree of semantic similarity. SNP 1 and SNP A do not share any terms directly; however, SNP 1’s “Term 1.1.1.1” and SNP A’s “Term 1.1.1.2” are both child terms of “Term 1.1.1” and so a similarity measure can be calculated based on this term. “Term 1.1.1” is the most informative common ancestor (MICA) between the two SNP annotation profiles, meaning they have a relatively high degree of similarity. The MICA of SNP 1 and SNP B is “Term 1”. Since this term is less informative than the MICA of SNP 1 and SNP A (lower IC value), the similarity score between SNP 1 and SNP B is lower. **b** Semantic similarity results for real data. Global similarity output for *cis-*eQTLs induced by 24-h IFN-γ is shown in the circos plot (*top left*). The top similarity links involving a specific SNP, rs11150589, are shown in the main circos plot, together with DAG plots showing the terms annotating each SNP. The genes modulated by the eQTL SNPs are given in *brackets*

The similarity between two SNPs is calculated based on 1) their annotation by EFO terms organised as a DAG, 2) specificity of terms, quantified by information content (IC) indicative of their frequency of annotation (including both original and inherited annotation), and 3) term–term similarity measured as IC at the MICA of two terms. Figure 9a illustrates the workflow and the key concepts behind SNP similarity analysis. The output is visualised as a circos plot, showing the SNP locations, and their pairwise similarity by coloured links. To help understand the similarity results, DAG plots are used to visualise the annotation profiles, with nodes coloured according to IC and shaped according to the type of annotation. In this toy example, SNP 1 is most similar to SNP C as they have the same annotation profile and share the highly informative Term.1.1.1.1. It is less similar to SNP A, as the MICA they share is a less informative parent term, and least similar to SNP B as the MICA is the root term. Figure 9b shows the similarity results when exploring *cis-*eQTLs induced by 24-h IFN-γ treatment. A circos plot displays the similarity results for all *cis-*eQTL SNPs, which can be reduced to display the similarity links involving a specific SNP, in this case rs11150589 (GWAS SNP in UC). The DAG plots clearly show why this SNP is most similar to rs10500264 (GWAS SNP in IBD), and has greater similarity to rs3957148 (GWAS SNP in MS) than rs2066807 (GWAS SNP in PSO). Together with knowledge of eQTL-containing genes such as *ITGAL cis-*regulated by rs11150589 and *CNPY2* by rs2066807 (Fig. 9b), disease profile-derived similarity between SNPs adds a new dimension to eQTL mapping interpretations. By identifying pairs of SNPs sharing the similar annotation/trait profiles, this piece of information can be used to select variants for follow-up functional studies such as from QTL mapping. SNP similarity measured in this way would be also useful in predicting physical interactions between genomic regions involving both SNPs, particularly when SNP annotations by EFO become more complete.

## Discussion

### Demanding issues addressed by XGR

In the current era of high-throughput genomics, the volume of data relating to complex human disease is growing at an unprecedented rate. The NHGRI-EBI GWAS Catalog contains 2546 studies at the time of writing (October 2016) [3], and there have been many success stories in terms of the identification of risk loci and the discovery of disease mechanisms. However, genomics has not yet nearly realised its full potential in this regard. In general, the generation of large datasets and their analysis through association studies are not the end goal of disease genomics, but instead represent a starting point for downstream interpretation, which aims to place preliminary results in a biological context. This post-GWAS stage benefits from the leveraging of multiple data sources and requires a general framework for the integration of the available knowledge and the application of appropriate methodologies to reveal the underlying information in a systematic way. XGR is created to meet this emerging need.

### Web-app user interface of XGR

All results described above are generated using the R package. To target users who are unfamiliar with R, we also develop a user-friendly web interface for each of the analyses supported by XGR (Fig. 1). In the web-app, users can simply paste gene or SNP lists of interest, choose an ontology or network, and specify parameters (or at default values). After submission, users can download, search, and explore the outputs in the form of various visuals.

### Generality of analyses supported by XGR

As well as software, XGR is also a resource incorporating diverse data types, thereby enabling comprehensive investigation of a gene or SNP set through enrichment, network, similarity, and annotation analysis. User input is not limited to the gene or SNP-centric data types. XGR can also be used to analyse genomic regions directly (Fig. 6), or indeed carry out enrichment analysis for any entity, e.g. protein domains (as demonstrated in the web-app). Overall, XGR is designed to be scalable, whilst also being efficient and effective.

### Uses and benefits of XGR

In the “Results” section, we demonstrate the intended uses of XGR to interpret three commonly encountered types of genomic summary data: gene sets resulting from differential expression studies; GWAS SNPs from GWAS summary data; and eQTL SNPs from eQTL summary data. In these use cases, we explore the genetic landscape of the immune system and immunological disorders, using differential expression and eQTL data for stimulated monocytes and the GWAS summary data for a dozen or so common diseases. These showcases are intended to give an overview of the workflow and functionality of XGR, while simultaneously showing the benefits of XGR to uncover interesting biology in real applications. For example, we find evidence for a link between the immune system and Parkinson’s disease when re-interpreting context-specific eQTL (Fig. 8). This is supported by a recent study suggesting that Parkinson’s disease may be considered as an autoimmune disease [41] with aging-induced changes in the immune system a potential contributor, and highlights the need for further work in this area. Another interesting finding is the disease vitiligo, overrepresented in analysis of eQTL but only involving activated monocytes; this is consistent with the hypothesis that vitiligo is triggered by cellular stress, danger signals, and innate immune activation [42]. Similarity analysis adds a new dimension in interpreting eQTL SNPs, not just showing their relevance to GWAS traits but also measuring how similar they are to each other in the meaning of trait profiles (i.e. ontology annotation profiles). Network analysis in XGR is unique in its power to identify SNP-modulated gene networks, defining disease subtypes based on GWAS SNPs (Fig. 3), and revealing shared and unique features across subtypes. The disease subtypes correspond well with the idea that immunological disorders form a spectrum from autoinflammatory to autoimmune based on clinical and mechanistic features [40]. It is generally recognised that pathophysiological mechanisms are shared across this disease spectrum to a greater or lesser extent. The analysis presented in this study, together with other studies leveraging the informativeness of current genetic data [7, 43, 44], helps to reveal the nature of these relationships, illustrating how cross-disease analysis can enhance opportunities for identifying central mediators as potential drug targets.

### Improved performance of XGR

We evaluate the performance of XGR in generating more informative results than conventional analyses. In particular, we show the necessity of respecting the ontology tree-like structure during enrichment analysis, either for genes or SNPs (Figs. 2 and 7). In the literature, the use of ontologies has gained popularity but is largely done without taking the structure itself into account (thus much less effective). We also show that XGR is able to perform cross-disease analysis. When coupled with annotation analysis (via nearby gene annotations or via co-localised functional genomic annotations), XGR is able to perform in-depth interpretation of the underlying genetic landscape of immunological diseases (Figs. 5 and 6). Therefore, XGR provides a single integrated solution to improve interpretation of genomic summary data for downstream biological discovery; this can also be seen from Table 2, which provides a comparison in terms of functionality and availability between XGR and other freely available tools, such as DAVID [45], GREAT [46], DEPICT [47], GOSemSim [48], GRAIL [49], dnet [36], and jActiveModule [50], to name but a few. This comparison also identifies a need for XGR to support other uses such as prioritisation, and to provide an online discussion/FAQ platform as the user base increases.

**Table 2:** Comparison to other commonly used tools

	Enrichment analysis		Annotation analysis		Similarity analysis		Network analysis		Availability		Prioritisation	Communication
	Support multiple ontologies	Respect ontology structure	Via gene annotation	Via genomic annotations	Between genes	Between SNPs	Gene-based	SNP-based	Package	Web-app	Gene-level prioritisation	Mailing list/FAQ platform
XGR	Yes	Yes	Yes	Yes	Yes	Yes	Yes	Yes	Yes	Yes	-	-
DAVID	Yes	No	-	-	-	-	-	-	No	Yes	-	Yes
GREAT	Yes	No	Yes	No	-	-	-	-	No	Yes	-	Yes
DEPICT	Yes	No	Yes	Yes	-	-	-	-	Yes	No	Yes	Yes
GOSemSim	-	-	-	-	Yes	No	-	-	Yes	No	-	-
GRAIL	-	-	-	-	-	-	No	Yes	No	Yes	Yes	-
dnet	Yes	Yes	-	-	-	-	Yes	No	Yes	No	-	-
jActiveModule	-	-	-	-	-	-	Yes	No	Yes	No	-	Yes

### Future development of XGR

We are actively engaged in, and have a long-term commitment to, ensuring XGR is updated and expanded on a regular basis (both functionality and data sources) as the field advances. For example, the built-in data include a number of structured ontologies, e.g. GO, DO, Human Phenotype Ontology, and EFO. The hierarchical nature of ontologies provides additional information concerning the relationships between terms, which we leverage to enhance downstream biological discovery and increase the informativeness of the outputs generated. SNP-level analysis supported in XGR is unique in its ontology tree-awareness through mapping of GWAS Catalog traits to EFO and the ability to calculate semantic similarity, but is currently restricted to use of this single ontology. As additional resources become available for orthogonal knowledge domains, these will be incorporated into XGR to expand its capacity for multi-layered investigation of genomic summary data. Other than the data expansion, future efforts will focus on increasing and enabling the user base (including deployment to community-driven genomics projects), evaluating predictive use of SNP similarity in chromosomal interactions (such as promoter interactomes [51]), and extending the network analysis to the genomic region level (such as differentially methylated regions).

## Conclusions

The publicly available XGR R package and web-app (Fig. 1) presented here provide a user-friendly, flexible, and powerful tool for the exploration and interpretation of genomic summary data. As the field of big data continues to expand and new resources become available, XGR will evolve alongside as an integrated solution for revealing underlying biological information.

## Availability and requirements

**Project name**: XGR

**Project home page (web-app)**: http://galahad.well.ox.ac.uk/XGR

**R package**: http://cran.r-project.org/package=XGR

**Operating system(s)**: Linux, Mac OS X, Windows

**Programming language**: R

**License**: GNU GPL

**Any restrictions to use by non-academics**: None.

## Additional files

Additional file 1: Comparison of three tests used for enrichment analysis. The tests compared are hypergeometric test, Fisher’s exact test, and binomial test. The DO enrichment analysis is applied to the same set of genes, namely differentially expressed genes induced by IFN-γ treatment of primary human monocytes [24]. (PDF 206 kb)
Additional file 2: Exploring the statistical test for enrichment through null simulations. The hypergeometric test is used for DO enrichment analysis applied to a set of genes, namely differentially expressed genes induced by IFN-γ treatment of primary human monocytes [24], identifying eight enriched terms (FDR <0.05). To estimate the chance of these enriched terms resulting from the real data that would be expected from a null distribution, we simulate a random set of genes (having the same number of genes as in the real data) for 10,000 times. Applying DO enrichment analysis to the simulated data, we count how often each enriched term is called significant under FDR <0.05. We also count how often each enriched term is called significant from the simulated data, but under the same or lower term-specific FDR (for example, 3.10E-05 for the term ‘viral infectious disease’). (PDF 58 kb)
Additional file 3: Estimating false positive rate for enrichments of genes of different sizes through null simulations. We use DGIdb gene druggable categories [30] for this purpose; there are a total of ~30 gene categories (thus computationally feasible), with gene members of different sizes. For each category, we simulate a random set of genes (having the same number as genes annotated by this category) for 20,000 times, and estimate how often (false positive rate) this category would be identified as enrichment (under different FDR cutoffs: <1E-1, <5E-2, <1E-2 and <5E-3) from the simulated data. **a** Histogram plot of FDR calculated from the simulated data, using the term ‘Tumor suppressor’ as an exemplar. **b** Dot plot of false positive rate (on the *x-axis*) for gene categories (ordered by the size of gene members on the *y-axis*). (PDF 565 kb)
Additional file 4: Justification of the 90 and 50% criteria used to remove redundant terms resulting from enrichment analysis. The pathway enrichment analysis is applied to the same set of genes (that is, differentially expressed genes induced by IFN-γ treatment of primary human monocytes [24]). **a** >90% of members in a redundant term that overlap with members in a more significant term. **b** >50% of members in a more significant term that overlap with members in a redundant term. (PDF 234 kb)
Additional file 5: Correlations of SNP similarity using best-matching (BM)-based methods. BM methods compared are average (*BM.average*), maximum (*BM.max*), and complete (*BM.complete*). SNP similarity analysis is applied to the same set of SNPs (*cis-*eQTLs) induced by IFN-γ treatment of primary human monocytes [24]. (PDF 129 kb)

## Abbreviations

AS: Ankylosing spondylitis
ATD: Autoimmune thyroid disease
BM: Best-matching
CEL: Celiac disease
CRO: Crohn’s disease
DAG: Directed acyclic graph
DO: Disease ontology
EFO: Experimental Factor Ontology
eQTL: Expression quantitative trait loci
FDR: False discovery rate
FWER: Family-wise error rate
GO: Gene Ontology
GWAS: Genome-wide association study
IBD: Inflammatory bowel disease
IC: Information content
IFN: Interferon
IL: Interleukin
LD: Linkage disequilibrium
MICA: Most informative common ancestor
MS: Multiple sclerosis
PSO: Psoriasis
RA: Rheumatoid arthritis
SLE: Systemic lupus erythematosus
SNP: Single-nucleotide polymorphism
T1D: Type 1 diabetes
UC: Ulcerative colitis
XGR: eXploring Genomic Relations
