# Supplementary figures and images for: XGR software for enhanced interpretation of genomic summary data, illustrated by application to immunological traits
Source: Genome Med. 2016 Dec 13;8:129. doi: 10.1186/s13073-016-0384-y (PMC5154134; doi:10.1186/s13073-016-0384-y)

# Enrichments under FDR < 0.05

*Hypergeometric test*

*Fisher's exact test*

*Binomial test*

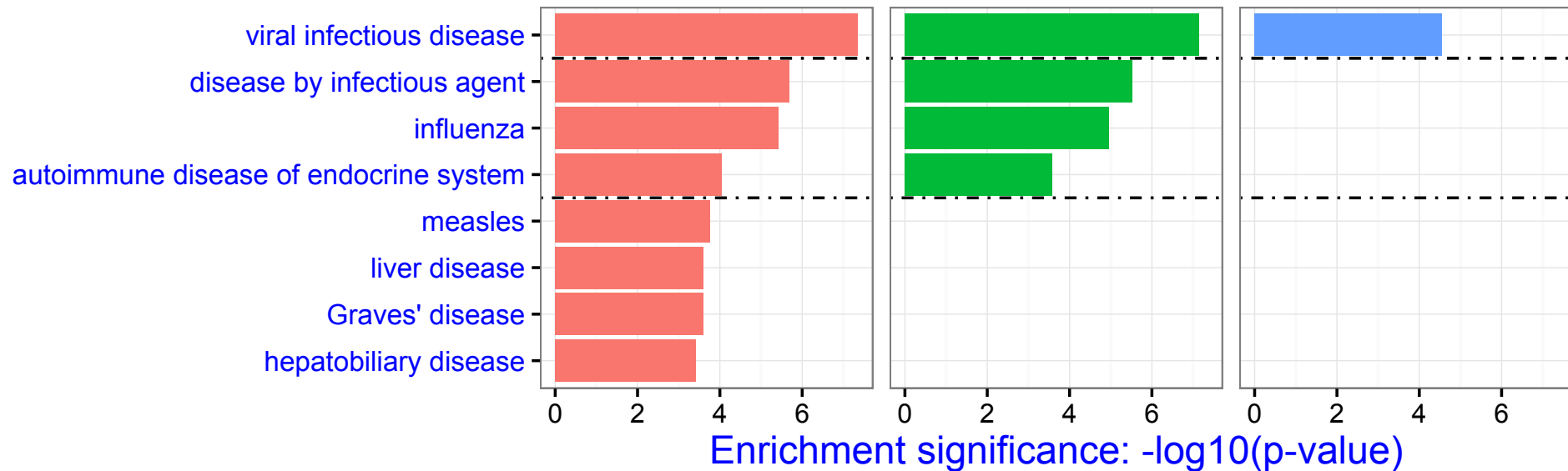

Supplement: Additional file 1: — Comparison of three tests used for enrichment analysis. The tests compared are hypergeometric test, Fisher’s exact test, and binomial test. The DO enrichment analysis is applied to the same set of genes, namely differentially expressed genes induced by IFN-γ treatment of primary human monocytes [24]. (PDF 206 kb) [file 13073_2016_384_MOESM1_ESM.pdf]

**A**

Histogram plot of FDR calculated from the 20,000 simulated data  
Tumor suppressor (n=716)

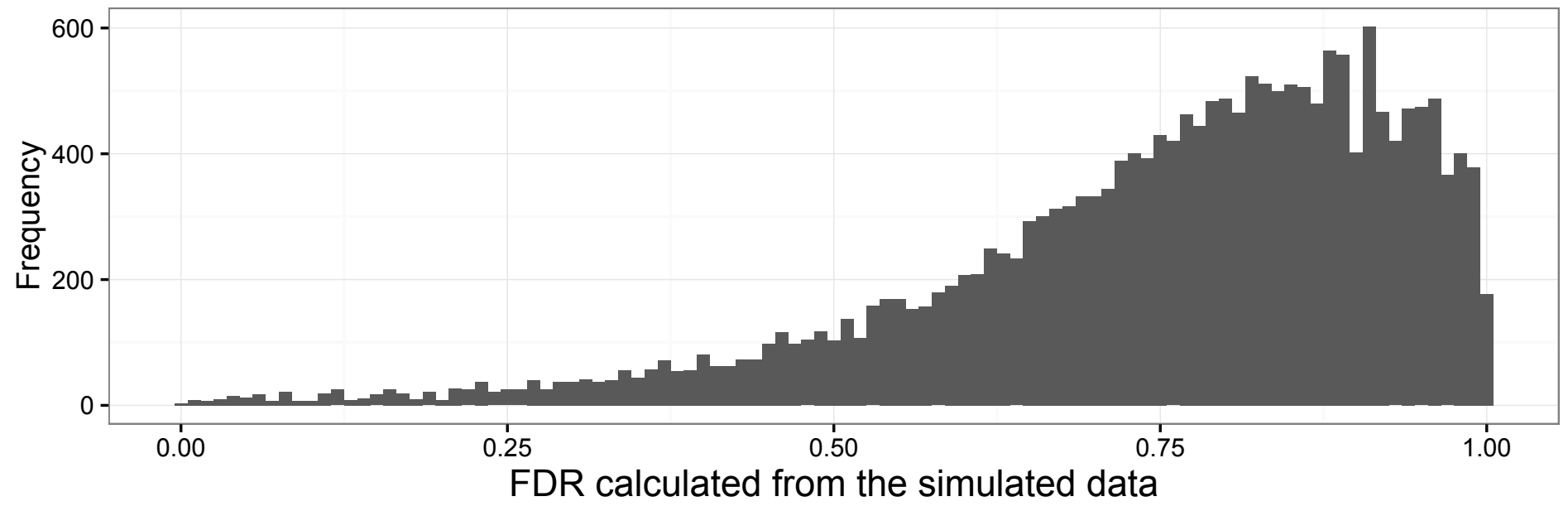**B**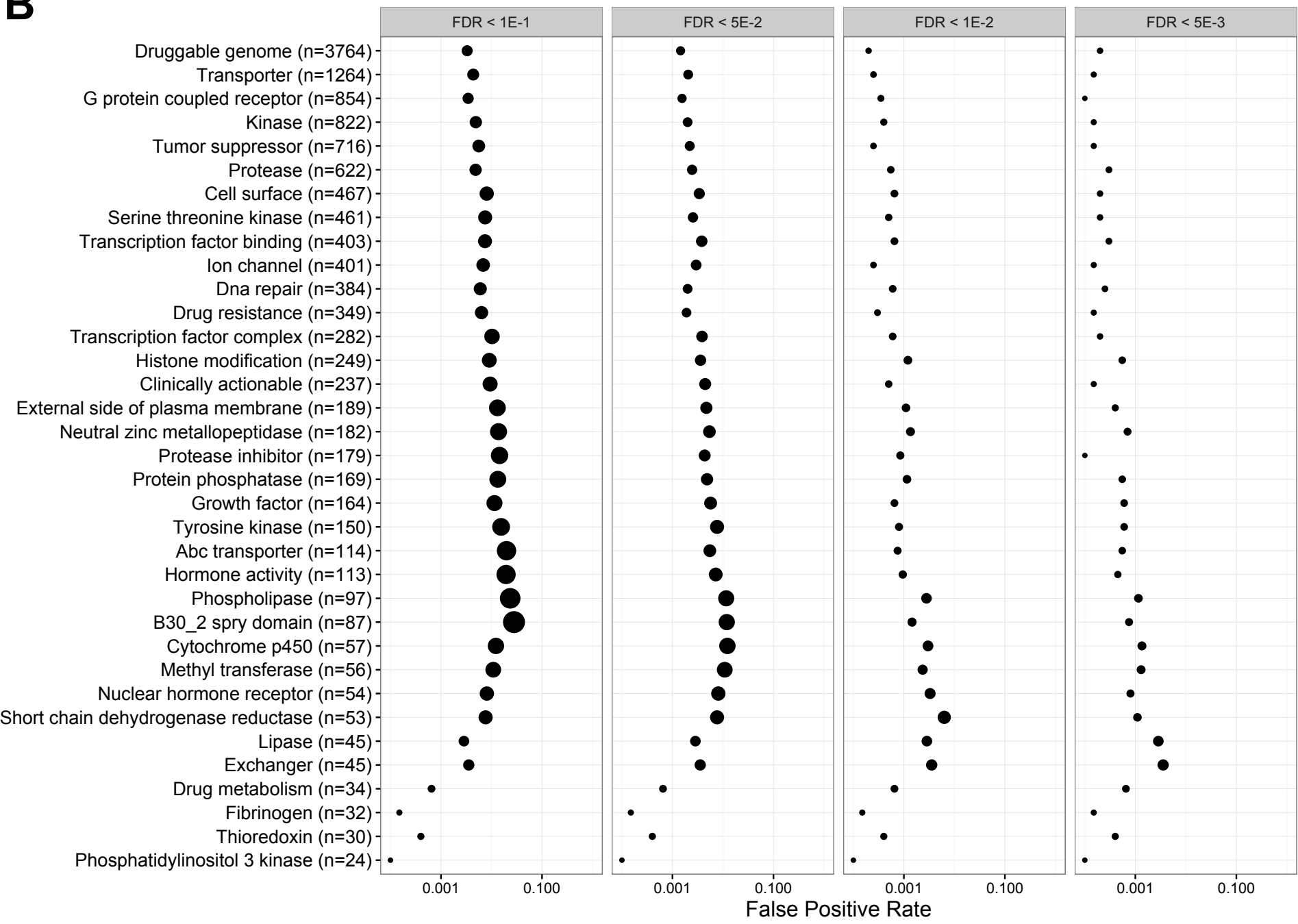

Supplement: Additional file 3: — Estimating false positive rate for enrichments of genes of different sizes through null simulations. We use DGIdb gene druggable categories [30] for this purpose; there are a total of ~30 gene categories (thus computationally feasible), with gene members of different sizes. For each category, we simulate a random set of genes (having the same number as genes annotated by this category) for 20,000 times, and estimate how often (false positive rate) this category would be identified as enrichment (under different FDR cutoffs: <1E-1, <5E-2, <1E-2 and <5E-3) from the simulated data. a Histogram plot of FDR calculated from the simulated data, using the term ‘Tumor suppressor’ as an exemplar. b Dot plot of false positive rate (on the x-axis) for gene categories (ordered by the size of gene members on the y-axis). (PDF 565 kb) [file 13073_2016_384_MOESM3_ESM.pdf]

**A**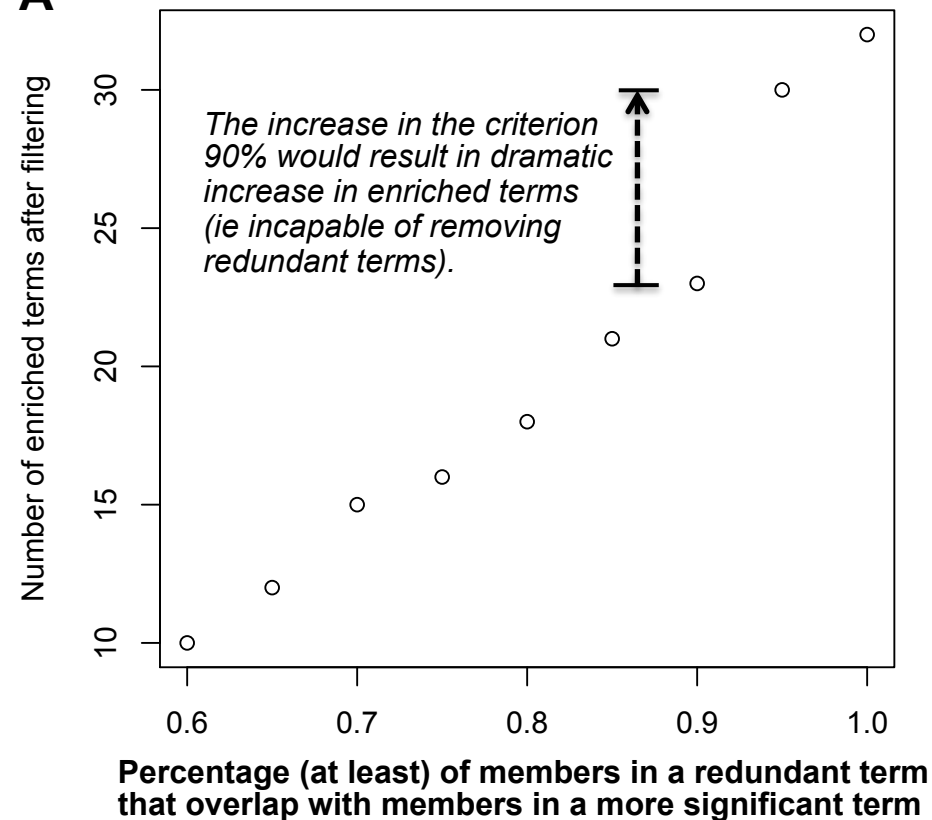**B**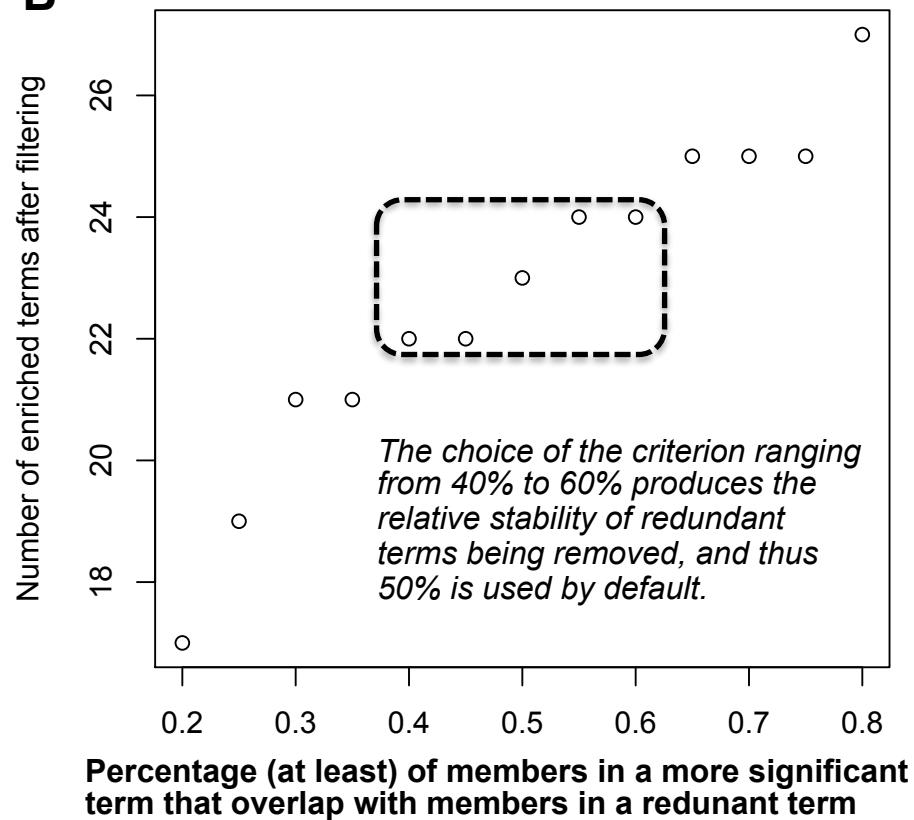

Supplement: Additional file 4: — Justification of the 90 and 50% criteria used to remove redundant terms resulting from enrichment analysis. The pathway enrichment analysis is applied to the same set of genes (that is, differentially expressed genes induced by IFN-γ treatment of primary human monocytes [24]). a >90% of members in a redundant term that overlap with members in a more significant term. b >50% of members in a more significant term that overlap with members in a redundant term. (PDF 234 kb) [file 13073_2016_384_MOESM4_ESM.pdf]

# Pearson Correlation

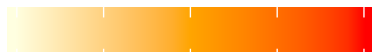

0.00 0.25 0.50 0.75 1.00

BM.max

1

BM.average

1

0.93

BM.complete

1

0.82

0.77

BM.complete

BM.average

BM.max

Supplement: Additional file 5: — Correlations of SNP similarity using best-matching (BM)-based methods. BM methods compared are average (BM.average), maximum (BM.max), and complete (BM.complete). SNP similarity analysis is applied to the same set of SNPs (cis-eQTLs) induced by IFN-γ treatment of primary human monocytes [24]. (PDF 129 kb) [file 13073_2016_384_MOESM5_ESM.pdf]
