# Supplementary material for: XGR software for enhanced interpretation of genomic summary data, illustrated by application to immunological traits
Source: Genome Med. 2016 Dec 13;8:129. doi: 10.1186/s13073-016-0384-y (PMC5154134; doi:10.1186/s13073-016-0384-y)
Supplement: Additional file 2: — Exploring the statistical test for enrichment through null simulations. The hypergeometric test is used for DO enrichment analysis applied to a set of genes, namely differentially expressed genes induced by IFN-γ treatment of primary human monocytes [24], identifying eight enriched terms (FDR <0.05). To estimate the chance of these enriched terms resulting from the real data that would be expected from a null distribution, we simulate a random set of genes (having the same number of genes as in the real data) for 10,000 times. Applying DO enrichment analysis to the simulated data, we count how often each enriched term is called significant under FDR <0.05. We also count how often each enriched term is called significant from the simulated data, but under the same or lower term-specific FDR (for example, 3.10E-05 for the term ‘viral infectious disease’). (PDF 58 kb) [file 13073_2016_384_MOESM2_ESM.pdf]

DO terms		Observed enrichments		Null simulations (10,000)	
ID	*Name*	*Z-score*	*FDR*	*How often called significant (FDR < 0.05)*	*How often called significant under the same or lower term-specific FDR*
DOID:934	viral infectious disease	5.35	3.10E-05	6 (0.06%)	0 (<0.01%)
DOID:0050117	disease by infectious agent	4.59	7.30E-04	12 (0.12%)	0 (<0.01%)
DOID:8469	influenza	4.41	8.80E-04	1 (0.01%)	0 (<0.01%)
DOID:0060005	autoimmune disease of endocrine system	3.65	1.60E-02	1 (0.01%)	0 (<0.01%)
DOID:8622	measles	3.39	2.40E-02	3 (0.03%)	0 (<0.01%)
DOID:409	liver disease	3.45	2.50E-02	4 (0.04%)	1 (0.01%)
DOID:12361	Graves' disease	3.38	2.50E-02	1 (0.01%)	0 (<0.01%)
DOID:3118	hepatobiliary disease	3.33	3.40E-02	7 (0.07%)	1 (0.01%)
